# An Artificial Intelligence-Based Model to Predict Pregnancy After Intrauterine Insemination: A Retrospective Analysis of 9501 Cycles

**DOI:** 10.3390/jpm15070308

**Published:** 2025-07-12

**Authors:** Jaume Minano Masip, Camille Grysole, Penelope Borduas, Isaac-Jacques Kadoch, Simon Phillips, Doina Precup, Daniel Dufort

**Affiliations:** 1 Département D’obstétrique et de Gynécologie, Faculté de Médecine, Université de Montréal, Montreal, QC H3T 1J4, Canadas.phillips@cliniqueovo.com (S.P.); 2 Department of Obstetrics and Gynecology, CHUM, Montreal, QC H2X0C1, Canada; 3 Division of Experimental Medicine, McGill University, Montreal, QC H4A 3J1, Canada; daniel.dufort@mcgill.ca; 4 Department of Medical Gynecology, Reproductive Medicine and Fertility Preservation, CHU Lille, Jeanne de Flandre Hospital, 59000 Lille, France; cgrysole@gmail.com; 5 Clinique Ovo, Montreal, QC H4P 2S4, Canada; 6 Department of Computer Science, McGill University, Montreal, QC H4A 3J1, Canada; 7 Child Health and Human Development Program, Research Institute of the McGill University Health Centre, Montreal, QC H3H 2R9, Canada; 8 Department of Obstetrics and Gynecology, McGill University, Montreal, QC H4A 3J1, Canada

**Keywords:** reproductive medicine, predictive model, intrauterine insemination, artificial intelligence, machine learning

## Abstract

**Background/Objectives:** Intrauterine insemination (IUI) is a common first-line approach in the treatment of numerous infertile couples, especially in cases of unexplained infertility. Its relatively low success rate, however, could benefit from the development of AI-based support tools to predict its outcome, thus helping the clinical management of patients undergoing IUI cycles. Our objective was to develop a robust and accurate machine learning model that predicts pregnancy outcomes following IUI. **Methods:** A retrospective, observational, and single-center study was conducted. In total, 3535 couples (aged 18–43 years) that underwent IUI between January 2011 and December 2015 were recruited. Twenty-one clinical and laboratory parameters of 9501 IUI cycles were used to train different machine learning algorithms. Accuracy of pregnancy outcome was evaluated by an area under the curve (AUC) analysis. **Results:** The linear SVM outperformed AdaBoost, Kernel SVM, Random Forest, Extreme Forest, Bagging, and Voting classifiers. Pre-wash sperm concentration, the ovarian stimulation protocol, cycle length, and maternal age were strong predictors of a positive pregnancy test following IUI (AUC = 0.78). Paternal age was found to be the worst predictor. **Conclusions:** Our Linear SVM model predicts a positive pregnancy outcome following IUI. Although this model shows value for the clinical management of infertile patients and informed decision-making by the patients, further validation using independent datasets is required prior to clinical implementation.

## 1. Introduction

Intrauterine insemination (IUI) is a common first-line approach in cases of unexplained infertility. This technique has a low incidence of complications and broadly addresses several conditions, including mild endometriosis, ovulation dysfunctions, and moderate male infertility, in addition to giving women without a male partner the opportunity to conceive. One of the notable advantages of IUI is the relatively low cost compared with other assisted reproductive technologies (ARTs), allowing couples to afford multiple cycles and increase their chances of success. The pregnancy rate per IUI cycle varies, ranging from 7% when oral stimulation agents are utilized to 12% when a recombinant follicle-stimulating hormone (FSH) is employed [1]. Several studies have focused on identifying prognostic factors that predict the success of IUI treatment in an effort to provide tools to manage patients’ expectations and orient them on personalized care pathways. Among these factors, the most relevant are total motile sperm count [2,3], female age [4], duration of infertility [5], and type of infertility [6]. Unfortunately, most models have been developed using small datasets and making use of a limited number of variables [7,8,9]. Small datasets specifically cause model overfitting, with little reproducibility when used with real-world data; this is especially problematic in ART due to the significant variations among patients, making it crucial to accurately predict outcomes for each individual. Additionally, using only a few parameters limits the model’s ability to understand the many aspects of reproductive health. Important factors like genetics, lifestyle, and environment might be missed, reducing the accuracy and usefulness of these models [10,11,12,13].

In recent years, artificial intelligence (AI) has shown great potential in helping with healthcare tasks like classifying medical images and predicting outcomes. Machine learning (ML) has been extensively used in ART to simplify tasks such as embryo quality prediction [14], prediction of the best FSH starting dose [15], or oocyte quality assessments [16]. However, human (in)fertility is a complex interplay of numerous biological, environmental, and psychosocial factors. Due to this, simpler predictive models have often struggled to account for this complexity. An area where ML can significantly contribute to the improvement in care is the prediction of IUI success. Although the individual machine learning techniques employed are well-established, their application to a comprehensive, large-scale dataset of well-characterized IUI cycles, coupled with a detailed feature importance analysis, can provide a robust and clinically actionable predictive model for pregnancy outcomes, addressing an unmet need in reproductive medicine.

In this study, we leveraged ML models to enhance the reliability and applicability of our predictive model. We utilized a larger dataset, enriched with a wide range of clinical factors. We assessed various models to determine which offered the highest predictive accuracy, and we also ranked patient features by their impact on model performance to enhance understanding of model mechanics. This analysis helped us to identify critical factors that improve predictions of pregnancy outcomes following IUI. We refer to our approach as “Smart IUI”, aimed at assisting clinicians in identifying couples who are most likely to benefit from IUI treatment.

## 2. Materials and Methods

### 2.1. Ethical Approval

Written informed consent was provided by all participants.

### 2.2. Study Design

This retrospective observational study analyzed data from 3535 couples aged 18–43 who underwent IUI between January 2011 and December 2015 at a single university-affiliated fertility center in Montreal, Canada. The dataset created for this study includes de-identified patient characteristics and clinical outcomes from 9501 IUI cycles. Twenty-one features were extracted for each cycle, including the male and female patient age, sperm quality parameters, number of previous IUI cycles, type of ovarian stimulation protocol, and number of days between menses and insemination or pregnancy (see Supplementary Table S1 for details). The database was pre-processed for consistency and clinical accuracy, and was then split into training, validation, and test sets. Hyperparameters were optimized using a stratified four-fold cross-validation to determine which models performed better, ultimately ranking the features used for the model.

### 2.3. Eligibility Criteria

Data were included in the study if the couple underwent at least one IUI cycle for any of the following indications: mild male factor infertility, mild endometriosis, ovulatory dysfunction, and unexplained infertility.

### 2.4. Ovarian Stimulation Protocols

The ovarian stimulation protocols involved oral agents, injectable gonadotropins, combination therapy, or natural cycles. Oral treatments, including clomiphene citrate (Clomid^®^, Sanofi-Aventis Canada, Laval, QC, Canada; 50–200 mg/day), letrozole (Femara^®^, Novartis Pharma Canada, Dorval, QC ,Canada; 2.5–5 mg/day), or tamoxifen (Nolvadex^®^, AstraZeneca Canada Inc., Mississauga, ON, Canada ; 20–60 mg/day), were administered for five days, starting from the third day of the menstrual cycle. A recombinant human follicle-stimulating hormone alone (Gonal F^®^, EMD Serono Canada, Mississauga, ON, Canada; Puregon^®^, Merck Canada Inc., Kirkland, QC, Canada), or in combination with a luteinizing hormone (Menopur^®^ or Repronex^®^, Ferring Canada, Toronto, ON, Canada), was administered at a dose ranging from 37.5 to 300 IU every one or two days until the trigger of ovulation, starting from the second or third day of the menstrual cycle. Protocols combining clomiphene citrate or letrozole with gonadotropins were employed in fewer cases. Patients undergoing natural cycles received no pharmacologic intervention.

All cycles were monitored using transvaginal ultrasound to assess the number and size of ovarian follicles as well as the endometrial thickness. When at least one antral follicle reached an average diameter of 18 mm, a subcutaneous injection of 250 μg of recombinant human chorionic gonadotropin (Ovidrel^®^, EMD Serono Canada, Mississauga, ON, Canada) was administered to trigger ovulation.

### 2.5. Sperm Analysis and Preparation

Fresh sperm samples were collected by masturbation following two to three days of sexual abstinence. Specimens were liquefied at 37 °C for 30 min prior to processing. Spermatozoa concentration, motility, and progression were microscopically analyzed (100× magnification) using 10 μL of semen in a Makler Chamber.

Sperm was prepared for IUI using density gradient centrifugation. A colloidal gradient consisting of 1 mL of 80% medium under 1 mL of 40% medium (Gynotec Sperm filter, Fertitech Canda Inc., Saint-Laurent, QC, Canada) was layered with a maximum of 4 mL of the sperm. After initial centrifugation at 400× *g* for 20 min, motile spermatozoa were transferred into 5 mL SpermWash^®^ (Gynotec Sperm wash, Fertitech Canada Inc., Saint-Laurent, QC, Canada) using a sterile Pasteur pipette for a second centrifugation at 100× *g* for 10 min. The resulting pellet was re-suspended in 0.5 mL SpermWash^®^ (Gynotec Sperm wash, Fertitech Canada Inc., Saint-Laurent, QC, Canada) for a post-wash analysis of sperm quality parameters and to calculate the number of motile spermatozoa inseminated (NMSI).

### 2.6. Intrauterine Insemination and Confirmation of Pregnancy

Intrauterine insemination (IUI) was performed 35–39 h after the ovulation trigger, using an insemination catheter (Mini space^®^, CCD, CooperSurgical, Toronto, ON, Canada). To support the luteal phase and promote implantation following IUI, patients were instructed to take 200 mg micronized progesterone (Prometrium^®^, Merck Canada Inc., Kirkland, QC, Canada) daily for 15 days following the procedure until 8 weeks of pregnancy. Patients under natural cycles or oral agents alone did not receive a supplementation regimen. Confirmation of pregnancy occurred two weeks post IUI through a patient-reported urine pregnancy test. Subsequently, an ultrasound was conducted at 7 weeks to validate the clinical pregnancy, which was considered to be the main outcome of the model.

### 2.7. Data Pre-Processing and Feature Normalization

**Figure 1:**
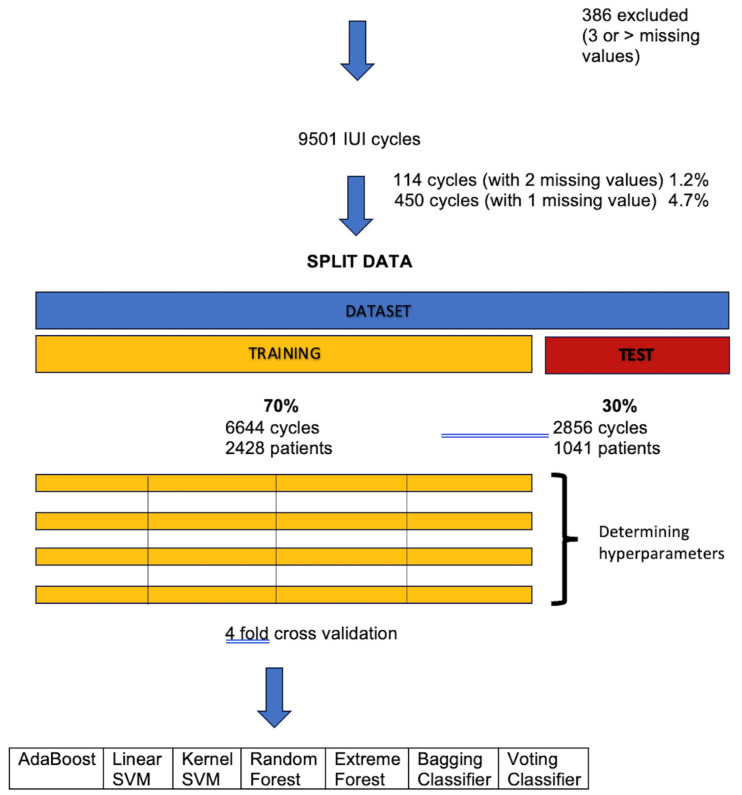
Overview of the data processing and machine learning pipeline used for predicting intrauterine insemination (IUI) success. The final dataset of 9501 cycles was split into a training set (70%,; 6644 cycles from 2428 patients) and a test set (30%; 2856 cycles from 1041 patients). Four-fold cross-validation was used within the training set for hyperparameter tuning. Multiple models were evaluated based on the area under the curve (AUC), including AdaBoost, Linear and Kernel Support Vector Machine (SVM), Random Forest, Extreme Forest, Bagging, and Voting classifiers.

To ensure the dataset’s consistency and suitability for analysis, we excluded cycles with data missing from three or more features. If only one or two features were missing, the feature’s median or mode was used to replace the missing value, as proposed by some authors [15]. Each cycle was associated with a label indicating whether the cycle resulted in clinical pregnancy, serving as the target variable for the predictive model (Figure 1).

Next, we tested six normalization methods (i.e., scale, normalization, robust scale, min–max, standard scaler, and PowerTransformer) to develop our ML model. The PowerTransformer yielded superior results by effectively transforming the data, aligning its distribution more closely with a Gaussian distribution, and was selected for further analysis [17]. The machine learning model was implemented in Python 3.11 using Scikit-learn for normalization [18].

The study dataset had four discrete, sixteen continuous, and one categorical variable/s (Supplementary Table S1). As the majority of machine learning algorithms are designed to handle only continuous or discrete variables, the categorical variables underwent one-hot encoding, transforming them into discrete variables. This approach created binary values for each category, providing input information for our model [19].

### 2.8. ML Model Training

The dataset was split into a training set (70% of the dataset; 6644 cycles from 2428 patients) and a testing set (30% of the dataset; 2856 cycles from 1041 patients) (Figure 1). Given that some couples underwent several IUI cycles, a stratified four-fold cross-validation was employed to avoid the overrepresentation of cycles from the same couple in each set (training or test). This approach balanced the data into four unique subsets of the same size. To optimize the models’ performance and improve robustness, we trained and validated each model on various combinations of preliminary hyperparameters with a Random Search [20]. For each combination, we used one of the datasets for testing and the remaining three for training. Finally, the average performance of the four models was recorded.

### 2.9. ML Model Selection

The performance of seven ML classification algorithms, including AdaBoost [21], Linear SVM [22], Kernel SVM [23], Random Forest [24], Extreme Forest [25], and Bagging [26] and Voting classifiers [27] was evaluated using the training set and four-fold cross-validation. These models were compared using the Scikit-learn library [18] to identify the model with the best performance. Model performance was quantified regarding the area under the curve (AUC) and ranked according to the model’s accuracy in predicting pregnancy following IUI.

### 2.10. Feature Ranking

Data patterns identified by predictive ML classifiers are often difficult to interpret as most classifiers are black boxes. However, the importance of the features used by the classifier can be ranked using recursive feature elimination (RFE) [28]. Given a classifier that assigns weights to features (e.g., Linear SVM), RFE selects the stronger features by recursively considering smaller and smaller sets of features. In this case, the Linear SVM was initially trained on all 21 features, and the weight of each feature was ranked using the feature importance attribute of the Linear SVM. Weaker features were eliminated one at a time until only one feature was left, and the model’s accuracy was improved. This approach identified the four features that are sufficient to predict a positive pregnancy outcome following IUI with maximum accuracy.

### 2.11. Data Availability

To ensure transparency and support reproducibility, the full-analysis scripts are available upon request. Interested researchers may obtain the code by contacting the first author via email at: jaume.minano.masip@umontreal.ca.

## 3. Results

### 3.1. Baseline Characteristics

A total of 9501 intrauterine insemination cycles from 3535 couples aged 18–43 years were included in the final analysis. The median ages for women and men were 34 (IQR 31–37 years) and 36 years old (IQR 32–40 years), respectively. The average IUI cycle duration was 27 ± 8 days, and the NMSI was 41 ± 37 M. The sperm concentration (88 ± 74 M/mL vs. 51 ± 38 M/mL; *p* < 0.01) and motility of progressive sperm (rapid and slow) were significantly improved after processing for IUI (83 ± 15% vs. 59 ± 20%; *p* < 0.01) compared with the initial specimen.

Four ovarian stimulation protocols were employed prior to IUI. Most patients received oral agents alone (79.58%; 7561 cycles) or in combination with exogenous gonadotropins (16.89%; 1605 cycles). Exogenous gonadotropins were employed alone in 216 cycles (2.27%), while natural cycles were used in 119 cycles (1.25%); the use of these protocols remained marginal regardless of how many IUIs the couple underwent. Oral agents were typically used alone in the first three insemination cycles (≥83% of cycles); however, from the fourth cycle onwards, they were often combined with exogenous gonadotropins to boost effectivity (Table 1). There was no significant difference between age groups; the distribution of cycles by age group exhibited similarity, with most cycles observed in patients aged 30 to 34 years (36.4%) (Supplementary Table S2).

In our cohort, 1230 (12.94%) IUI cycles resulted in a positive pregnancy test. Pregnancy rates declined with the first three insemination cycles (14.38%,12.43%, and 10.26%, respectively) but stabilized from the fourth cycle onwards (13%). Statistically significant differences were observed between different IUI attempts, as shown in Table 1. The cumulative pregnancy rate after four IUI cycles was 31.83%.

### 3.2. ML Model Selection

We tested various machine learning models, such as AdaBoost, Linear SVM, Kernel SVM, Random Forest, Extreme Forest, Bagging and Voting classifiers, on the study dataset to predict pregnancy outcomes. The performance of each model is summarized in Table 2. Among these models, the Linear SVM model outperformed the others, achieving the highest score of 0.76 across the four-fold cross-validation and test sets. This score measures how accurately a model predicts outcomes, with higher scores indicating better predictions.

Although the Linear SVM model performed well on both the training data and unseen test data, other models like the Extreme Random Forest, Bagging classifier, and Voting classifier performed well on the training data but their performance dropped when tested on new data.

### 3.3. Feature Ranking

We applied the recursive feature elimination (RFE) technique to refine our approach to predict pregnancy outcomes. RFE helps identify the most impactful features by iteratively removing the least important ones, thereby potentially increasing the accuracy of the machine learning model. Table 3 illustrates the results of feature ranking in our study using the Linear SVM model. Key features such as the pre-wash sperm concentration, type of ovarian stimulation received by the patient, cycle length, and maternal age were identified as the top contributors to our predictive model (Table 3 and Table S1).

Pre-wash sperm concentration emerged as the top-ranked predictive feature, suggesting that the quality of the raw semen sample plays a pivotal role in determining IUI success. Other sperm parameters, such as post-wash sperm A + B + C motility, post-wash concentration, and various individual motility categories (A, B, C, and D), showed lower but still measurable contributions to prediction accuracy. However, none of these features surpassed the influence of pre-wash concentration, which remained the only sperm-related variable among the top four predictors retained after recursive feature elimination.

Table 2 shows the benefits of RFE. By only utilizing the top 4 features identified by RFE rather than the entire set of 21 features, the SVM classifier reached a higher area under the curve (AUC) score of 0.78. This approach might appear counterintuitive but it underscores how focusing on fewer, more relevant features can boost model performance, as opposed to using a larger number of less relevant features, which can introduce noise and degrade performance.

The performance of all the machine learning classification algorithms was then evaluated using the following two distinct feature sets: “All”, described in the previous chapter, and “Top 4” (Supplementary Table S3). For the “All” feature set, the Linear SVM model achieved the highest area under the curve (AUC) at 0.76 ± 0.04, accompanied by a recall of 0.73 ± 0.01 and an F1 score of 0.76 ± 0.10. In contrast, the other models, including AdaBoost, Kernel SVM, Random Forest, Extreme Forest, and Bagging and Voting classifiers, exhibited AUC values ranging from 0.53 ± 0.05 to 0.57 ± 0.03. When utilizing the “Top 4” feature set, the Linear SVM model further improved its performance, reaching an AUC of 0.78 ± 0.04, with a recall of 0.77 ± 0.02 and an F1 score of 0.78 ± 0.04. The performance of the other models with the “Top 4” feature set remained within a similar range as the “All” feature set, with AUC values between 0.54 ± 0.04 and 0.58 ± 0.05.

## 4. Discussion

Machine learning models predicting the success of IUI have been extensively investigated as understanding the factors responsible for its success and using them to build reliable models could greatly help in clinical counselling and decision-making.

Our study demonstrated that the Linear SVM model was superior to the seven other ML models for predicting the event of pregnancy following IUI, with an AUC of 0.78. The extensive dataset we used, including 21 clinical features from 3535 couples that underwent a total of 9501 IUI cycles, allowed us to obtain a robust AUC that is suitable for use in clinical practice for both consultation and decision-making. Other studies provided similar predictions but possess limitations compared with the present work (Supplementary Table S4). Some studies focused on infertility diagnosis, semen parameters, and ovarian stimulation regimens [29,30]. However, several encountered challenges due to limited dataset sizes [31] and the limited number of features analyzed, which exacerbates issues related to overfitting in ML models [32]. Overfitting occurs when a model demonstrates strong predictive performance during training but struggles to perform when applied to an independent test dataset. Models characterized by significant overfitting often exhibit a reduced ability to generalize their findings, thus compromising their overall usefulness.

The factors with the highest predictive value reported in the literature often differ, with a few parameters consistently showing a positive predictive value, while others, such as sperm motility and morphology, showing conflicting results.

We found that 17 of the 21 clinical features in our database, including paternal age, proved to be redundant and/or did not significantly contribute to the predictive model. Feature ranking by RFE highlighted that the sperm concentration before processing, type of ovarian stimulation protocol, cycle length, and maternal age enhanced the accuracy and provided valuable insights into the functionality of our model. In this study, some may have had subpar performance because of common underlying IUI patient factors, including mild male factor or idiopathic infertility. To overcome this issue, each feature was treated as an independent predictor of overall pregnancy without assessing the relationships between these features [30].

The superior performance of the Linear SVM model, particularly with the reduced “Top 4” feature set (AUC = 0.78 ± 0.04), highlights its potential as a robust predictive tool for pregnancy outcomes following IUI. This finding suggests that a small set of key features can effectively capture the patterns influencing IUI success, aligning with the principle of parsimony in model development, as also indicated in different studies [33]. The marginal improvement in the AUC when moving from the “All” feature set to the “Top 4” for the Linear SVM indicates that feature selection can not only simplify the model but also enhance its predictive accuracy by reducing noise or irrelevant variables. This is a crucial aspect for clinical applicability as models relying on fewer, yet highly predictive, features are often more interpretable and easier to implement in real-world settings. The comparatively lower performance of other models, such as AdaBoost and Random Forest, highlights the suitability of the Linear SVM for this specific dataset and prediction task.

Our findings highlight the predictive importance of pre-wash sperm concentration, which aligns with prior clinical studies highlighting total motile sperm count as a key determinant of IUI success. The prominent role of this parameter in our model suggests that the initial quality of the semen sample retains prognostic significance, even after processing steps such as density gradient centrifugation. Interestingly, although several post-wash sperm parameters were included in the feature ranking, they did not further enhance prediction. This may reflect the effect of laboratory sperm preparation, which reduces variability among samples. It is also worth noting that motility indices (such as A + B + C motility) showed a moderate ranking, suggesting that aggregate motility metrics may better capture fertilization potential than isolated motility classes.

Although the literature extensively documents the negative effect of advanced maternal age on fertility [29] and the positive impact of a higher sperm concentration, the impact of advanced paternal age remains controversial. One study reported no discernible influence of paternal age on pregnancy outcomes when stratified for maternal age, despite declines in semen volume, concentration, and motility with advancing paternal age [34]. Our findings support that sperm concentration remains a key factor in achieving pregnancy, even with advanced paternal age.

There is an emerging interest in the influence of the follicular phase duration on the likelihood of achieving pregnancy through IUI. The follicular phase length has been associated with an increase in the chance of pregnancy of 6% for each additional day of the follicular phase [35]. Our study confirms and extends these findings, indicating that a longer follicular phase length leads to improved outcomes. This trend might be attributed to obtaining more mature follicles before IUI. Other researchers have explored the impact of anti-Müllerian hormone (AMH) levels and antral follicle count (AFC) as potential factors. Although a positive correlation was identified between AMH and pregnancy, no such correlation was found for AFC [36]. Considering this discovery, future studies should incorporate both features to assess their combined predictive value.

Importantly, in nearly 80% of IUI cycles, ovarian stimulation protocols involved oral agents. This observation reflects the established clinical practice at our facility and was consistent with patterns reported in other studies [37]. Throughout the five-year duration of this study, the practice of IUI remained consistent, with no significant modifications observed. This ensured that the prognostic factors under investigation were not affected or influenced over time.

Finally, effectively addressing the challenge of imbalanced data in machine learning is crucial to prevent the model from displaying bias toward the majority class. Given the substantial imbalance in our dataset, where only 12.9% of cycles resulted in successful pregnancies, we evaluated various models. To address the imbalance in our dataset, we chose the AUC as our primary evaluation metric because it effectively assesses a model’s ability to distinguish between different outcomes, such as predicting whether a pregnancy will be successful or not, regardless of the disproportionate sample sizes. Additionally, we implemented RFE to systematically identify and retain only the most impactful features, thereby enhancing the model’s predictive accuracy and robustness by focusing on the most relevant data.

### Limitations and Future Work

Our study possesses certain limitations. First, outcomes of clinical significance such as multiple pregnancies and live birth rates were unavailable in the dataset. Additionally, we lacked external validation, which was due to the limitations in data access because this study was developed in a single setting. This might have introduced biases and over- or underrepresented certain features, diminishing generalizability. Finally, we considered 21 features that were most likely to influence the observed outcome based on the known literature. However, we could have omitted some features that would have significantly improved the model. Looking for future directions, although this model shows value for the clinical management of infertile patients and informed decision-making by the patients, external validation using independent datasets is of paramount importance for a broader clinical implementation and generalizability, and represents the most immediate step to be undertaken, possibly by performing multi-center trials that could target the most diverse populations (and possibly use an increased number of features) to provide the basis for the wider implementation of such a method.

## 5. Conclusions

The Linear SVM model accurately predicted whether patients could achieve a clinical pregnancy following IUI, with an AUC of 0.78 when the dataset used contained features of the sperm concentration before processing for insemination, type of ovarian stimulation, cycle length, and maternal age. Based on recursive feature elimination, paternal age was irrelevant for the predictive model. Our model could become a valuable tool for clinicians who are counselling couples facing infertility after successful external validation. Predicting whether patients can achieve pregnancy with IUI will improve informed decision-making.

## Figures and Tables

**Table 1:** Ovarian stimulation protocols and pregnancy outcomes by history of intrauterine insemination cycle and stratified by IUI attempt. Statistical differences were found with an ANOVA followed by Tukey’s HSD. Data are presented as the number of cycles (n) and proportion (%) of the total 9501 IUI cycles analyzed (%). IUI: intrauterine insemination.

IUI Attempt	Total Number of Cycles	Ovarian Stimulation Protocol				Positive Pregnancy Test
		Oral Agents	Exogenous Gonadotropins	Combined Therapy	Natural Cycles	
1	3559 (37.45%)	2954 (83.00%)	82 (2.30%)	469 (13.20%)	54 (15.10%)	512 (14.38%)
2	2614 (27.51%)	2236 (85.50%)	46 (1.76%)	296 (11.32%)	36 (1.37%)	325 (12.43%)
3	1841 (19.37%)	1576 (85.60%)	32 (1.70%)	217 (11.80%)	16 (0.80%)	189 (10.26%)
4	774 (8.14%)	420 (54.26%)	28 (3.60%)	317 (40.95%)	9 (1.10%)	107 (13.82%)
5	713 (7.50%)	376 (52.73%)	28 (3.90%)	305 (42.80%)	4 (0.50%)	97 (13.60%)
Statistically significant differences were found between IUI 1 and IUI 2 (*p* = 0.001), IUI 1 and IUI 3 (*p* = 0.001), IUI 2 and IUI 3 (*p* = 0.001), IUI 2 and IUI 4 (*p* = 0.046), IUI 3 and IUI 4 (*p* = 0.001), and IUI 3 and IUI 5 (*p* = 0.001). No significant differences were observed between IUI 1 and IUI 4 (*p* = 0.488), IUI 1 and IUI 5 (*p* = 0.235), IUI 2 and IUI 5 (*p* = 0.163), and IUI 4 and IUI 5 (*p* = 0.9).						

**Table 2:** Results of models’ performance. The first column, labelled “Model”, lists the seven different models used in the study. The second column represents the hyperparameters; each model employed distinct parameters that were adjusted during training to enhance performance. The optimal hyperparameters for each model are detailed in the table. The third column denotes the time required to compute each model. Lastly, the fourth column, labelled “AUC”, displays the accuracies obtained using the 21 features in the first three columns (cross-validation (CV), training, and test), while “Top 4” indicates accuracies using recursive feature elimination (RFE). Note: The top-performance model is shown in bold. Six of the seven methods performed only marginally better than a 0.5 AUC score, which resembles random guessing. AUC: area under the curve.

Model		Parameters		Time (min)	AUC			
					21 Features			Top 4
					CV	Train	Test	
1	AdaBoost	Learning rate	1.0	14.7	0.585 ± 0.045	0.628 ± 0.02	0.57 ± 0.03	0.58 ± 0.05
		N_estimators	500					
2	**Linear SVM**	C	0.001	4.8	0.760 ± 0.045	0.790 ± 0.06	**0.76** ± **0.04**	**0.78** ± **0.04**
		Class weight	1:10					
3	Kernel SVM	Kernel rbf Gamma 0.001 Class weight 1:10 C: 1000		52.1	0.584 ± 0.057	0.676 ± 0.042	0.56 ± 0.05	0.57 ± 0.05
4	Random Forest	N_estimators 50 Max_depth 15 Bootstrap true Min_samples_split 0.1 Min_samples_leaf 0.01 Max_features none Class_weight: balanced_subsample		32.4	0.597 ± 0.044	0.689 ± 0.056	0.56 ± 0.03	0.58 ± 0.04
5	Extreme Forest	N_estimators 200 Max_depth none Bootstrap true Min_samples_split 2 Min_samples_leaf 10 Max_features auto Class_weight 1.25 Oob_score true		49.9	0.590 ± 0.054	0.872 ± 0.045	0.55 ± 0.04	0.56 ± 0.06
6	Bagging Classifier	N_estimators 400 Max_samples 0.25 Max_features 0.1 Bootstrap true Oob_score true		76.5	0.576 ± 0.040	0.883 ± 0.043	0.53 ± 0.05	0.54 ± 0.04
7	Voting Classifier	C 0.01 W 1:10		28.8	0.579 ± 0.047	0.853 ± 0.034	0.56 ± 0.05	0.57 ± 0.05

**Table 3:** Ranking of predictive features for intrauterine insemination (IUI) success based on their contribution to model accuracy. Definitions of all the features are presented in Supplementary Table S1. Recursive feature elimination (RFE) is a systematic process used for feature ranking that hinges on a model’s predictive accuracy. The procedure starts with a model fully trained on all available features. Using a support vector machine, the method assesses each feature’s contribution to model performance, and the least important feature is removed. The model is then retrained on this pruned feature set, and the cycle repeats. The importance of each feature is measured using the mean decrease in accuracy metric. This method assesses how much the accuracy of the model drops when the values of a feature are randomly shuffled, breaking its association with the target outcome. The elimination process continues if further removals lead to a significant performance degradation. The retained set of features, deemed the most critical for accurate predictions, is expected to balance simplicity and predictive power, enabling the model to perform effectively on new, unseen data. A (rapid progressive), B (slow progressive), C (non-progressive), and D (immotile) are combined to provide motility scores (e.g., A + B + C) and the NMSI (number of motile sperm inseminated).

Rank	Feature	Accuracy
1	Pre-wash sperm concentration	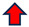
2	Ovarian stimulation	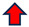
3	Cycle length	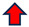
4	Maternal age	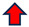
5	Post-wash sperm A + B + C	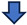
6	IUI history	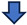
7	Post-wash sperm C	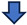
8	Post-wash sperm concentration	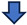
9	Pre-wash sperm B	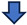
10	Post-wash sperm C	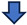
11	Post-wash sperm A + B	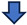
12	Pre-wash sperm A	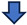
13	Pre-wash sperm D	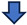
14	Pre-wash sperm C	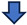
15	NMSI	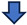
16	Pre-wash sperm A + B + C	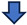
17	Pre-wash sperm A + B	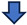
18	Number of days between menses and pregnancy test	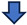
19	Post-wash sperm B	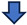
20	Post-wash sperm A	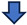
21	Paternal age	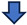

## Data Availability

The data supporting this study are not publicly available due to privacy and ethical restrictions.

## Supplementary Materials

The following supporting information can be downloaded at: https://www.mdpi.com/article/10.3390/jpm15070308/s1, Table S1: Description of the study dataset features. Table S2: Distribution of female patients undergoing IUI by age group. The majority of patients were between 30–34 years old (36%), followed by those aged 35–39 years (32%). Table S3 Performance metrics of various machine learning models for predicting IUI success using all available features and the top 4 selected features. Metrics reported include area under the ROC curve (AUC), recall, and F1 score with corresponding standard deviations based on cross-validation. Linear SVM consistently outperformed other models across both feature sets, particularly in AUC and F1 score. Table S4 Summary of selected studies evaluating predictive models for intrauterine insemination (IUI) success. The table compares datasets, number of features, analytical methods used, and main limitations. References [38,39,40,41,42] are cited in the supplementary materials.

## References

[1] Ayeleke R.O., Asseler J.D., Cohlen B.J., Veltman-Verhulst S.M. (2020). Intra-uterine insemination for unexplained subfertility. Cochrane Database Syst Rev..

[2] Jeong M., Kim S.K., Kim H., Lee J.R., Jee B.C., Kim S.H. (2021). Predictive value of sperm motility before and after preparation for the pregnancy outcomes of intrauterine insemination. Clin. Exp. Reprod. Med..

[3] Grysole C., Phillips S., Minano J., Velez M.P., Antaki R., Sylvestre C., Bissonnette F., Kadoch I.J. (2021). Series of 9386 IUI Cycles: The Impact of the Number of Motile Spermatozoa Inseminated Varies According to the Female Age. J. Vitr. Fertil..

[4] Yu C., Bai L., Mei-Zhou J., Yu-Wang X., Chen L., Zhang J. (2024). Analysis of factors associated with IUI pregnancy outcomes in elderly and young patients. BMC Womens Health.

[5] Huang C., Shi Q., Xing J., Yan Y., Shen X., Shan H., Sun H., Mei J. (2024). The relationship between duration of infertility and clinical outcomes of intrauterine insemination for younger women: A retrospective clinical study. BMC Pregnancy Childbirth.

[6] He W., Chen S., Huang J., Zhang X., Hu L., Xue Z., Qiu Y. (2022). Association Between Type of Infertility and Live Birth in Couples With a Single Intrauterine Insemination Resulting in Pregnancy: A Propensity Score Matching Cohort Study. Front. Endocrinol..

[7] Thijssen A., Creemers A., Van der Elst W., Creemers E., Vandormael E., Dhont N., Ombelet W. (2017). Predictive value of different covariates influencing pregnancy rate following intrauterine insemination with homologous semen: A prospective cohort study. Reprod. Biomed. Online.

[8] Danhof N.A., van Eekelen R., Repping S., Mol B.W.J., van der Veen F., van Wely M., Mochtar M.H., SUPER Study group (2019). Follicle stimulating hormone or clomiphene citrate in intrauterine insemination with ovarian stimulation for unexplained subfertility: A role for treatment selection markers?. Reprod. Biomed. Online.

[9] Ejzenberg D., Callado G.Y., de Oliveira Gomes T.J., Cavalcanti G.S., Soares J.M., Baracat E.C., Monteleone P.A. (2025). A new accurate model to assess intrauterine insemination success based on clinical parameters: Optimizing fertility treatment. Int. J. Gynaecol. Obstet..

[10] Bagherian E., Jokari S., Borjian Boroujeni P., Haratian K., Sabbaghian M., Mohseni Meybodi A. (2024). Role of genetic variations and protein expression of β-Microsemino protein in intrauterine insemination outcome of unexplained infertile men: A case-control study. Int. J. Reprod. Biomed..

[11] Legro R.S., Hansen K.R., Diamond M.P., Steiner A.Z., Coutifaris C., Cedars M.I., Hoeger K.M., Usadi R., Johnstone E.B., Haisenleder D.J. (2022). Reproductive Medicine Network. Effects of preconception lifestyle intervention in infertile women with obesity: The FIT-PLESE randomized controlled trial. PLoS Med..

[12] Luo Y., Wu S., Yuan J., Zhou H., Zhong Y., Zhang M., Li Q., Xu X., Sun X., Zhu D. (2021). Evaluation of Prognostic Factors for Clinical Pregnancy Rate Following Artificial Insemination by Husband in the Chinese Population. Front. Med..

[13] Xu R., Zhong Y., Li R., Li Y., Zhong Z., Liu T., Wang Q., Lv Z., Huang S., Duan Y.G. (2023). Association between exposure to ambient air pollution and semen quality: A systematic review and meta-analysis. Sci. Total Environ..

[14] Salih M., Austin C., Warty R.R., Tiktin C., Rolnik D.L., Momeni M., Rezatofighi H., Reddy S., Smith V., Vollenhoven B. (2023). Embryo selection through artificial intelligence versus embryologists: A systematic review. Hum. Reprod. Open.

[15] Correa N., Cerquides J., Arcos J.L., Vassena R. (2022). Supporting first FSH dosage for ovarian stimulation with machine learning. Reprod. Biomed. Online.

[16] Fjeldstad J., Qi W., Mercuri N., Siddique N., Meriano J., Krivoi A., Nayot D. (2024). An artificial intelligence tool predicts blastocyst development from static images of fresh mature oocytes. Reprod. Biomed. Online.

[17] Shi X., Prins C., Van Pottelbergh G., Mamouris P., Vaes B., De Moor B. (2021). An automated data cleaning method for Electronic Health Records by incorporating clinical knowledge. BMC Med. Inform. Decis. Mak..

[18] Pedregosa P., Varoquaux G., Gramfort A., Michel V., Thirion B., Grisel O., Blondel M., Prettenhofer P., Weiss R., Dubourg V. (2011). JMLR 12 Scikit-learn: Machine Learning in Python. J. Mach. Learn. Res..

[19] Kuhn M., Johnson K. (2013). Applied Predictive Modeling.

[20] Bergstra J., Bengio Y. (2012). Random Search for Hyper-Parameter Optimization. J. Mach. Learn. Res..

[21] Yoav F., Robert E.S. (1997). A Decision-Theoretic Generalization of On-Line Learning and an Application to Boosting. J. Comput. Syst. Sci..

[22] Cristianini N., Shawe-Taylor J. (2000). Support Vector Machines. An Introduction to Support Vector Machines and Other Kernel-Based Learning Methods.

[23] Khyathi G., Indumathi K.P., Hasin J., Siluvai S., Krishnaprakash G. (2025). Support Vector Machines: A Literature Review on Their Application in Analyzing Mass Data for Public Health. Cureus.

[24] Couronné R., Probst P., Boulesteix A.L. (2018). Random forest versus logistic regression: A large-scale benchmark experiment. BMC Bioinform..

[25] Geurts P., Ernst D., Wehenkel L. (2006). Extremely Randomized Trees. Mach. Learn..

[26] Zhang C., Ma Y. (2012). Ensemble Machine Learning: Methods and Applications.

[27] Raschka S. (2015). Python Machine Learning.

[28] Kursa M., Rudnicki W. (2010). Feature Selection with Boruta Package. J. Stat. Softw..

[29] Starosta A., Gordon C.E., Hornstein M.D. (2020). Predictive factors for intrauterine insemination outcomes: A review. Fertil. Res. Pract..

[30] Huang X., Sun Q., Tang X., Li M., Zhou C., Cheng X., Yao B., Chen L. (2023). Factors Influencing the Pregnancy Outcome of Intrauterine Insemination and Follow-up Treatment. J. Hum. Reprod. Sci..

[31] Ghaffari F., Sadatmahalleh S.J., Akhoond M.R., Eftekhari Yazdi P., Zolfaghari Z. (2015). Evaluating The Effective Factors in Pregnancy after Intrauterine Insemination: A Retrospective Study. Int. J. Fertil. Steril..

[32] Bengio Y., Ian G., Aaron C. (2017). Deep Learning.

[33] Moon M., Nakai K. (2016). Stable feature selection based on the ensemble L1-norm support vector machine for biomarker discovery. BMC Genom..

[34] Farabet C., Pirtea P., Benammar A., De Ziegler D., Marchiori C., Vallée A., Ayoubi J.M. (2024). The impact of paternal age on cumulative assisted reproductive technology outcomes. Front. Med..

[35] Bakkensen J.B., Christou G., Dimitriadis I., James K., Souter I. (2020). The effect of follicular phase length on cycle outcomes and endometrial development in gonadotrophin ovarian stimulation/intrauterine insemination cycles. Reprod. Biomed. Online.

[36] Moro F., Tropea A., Scarinci E., Leoncini E., Boccia S., Federico A., Alesiani O., Lanzone A., Apa R. (2016). Anti-Müllerian hormone concentrations and antral follicle counts for the prediction of pregnancy outcomes after intrauterine insemination. Int. J. Gynaecol. Obstet..

[37] Diamond M.P., Legro R.S., Coutifaris C., Alvero R., Robinson R.D., Casson P., Christman G.M., Ager J., Huang H., Hansen K.R. (2015). Letrozole, Gonadotropin, or Clomiphene for Unexplained Infertility. N. Engl. J. Med..

[38] Goldman R.H., Batsis M., Petrozza J.C., Souter I. (2014). Patient-specific predictions of outcome after gonadotropin ovulation induction/intrauterine insemination. Fertil. Steril..

[39] Erdem M., Erdem A., Mutlu M.F., Ozisik S., Yildiz S., Guler I., Karakaya C. (2016). The impact of sperm morphology on the outcome of intrauterine insemination cycles with gonadotropins in unexplained and male subfertility. Eur. J. Obstet. Gynecol. Reprod. Biol..

[40] Lemmens L., Kos S., Beijer C., Brinkman J.W., van der Horst F.A., van den Hoven L., Kieslinger D.C., van Trooyen-van Vrouwerff N.J., Wolthuis A., Hendriks J.C. (2016). Predictive value of sperm morphology and progressively motile sperm count for pregnancy outcomes in intrauterine insemination. Fertil. Steril..

[41] Ranjbari S., Khatibi T., Vosough Dizaji A., Sajadi H., Totonchi M., Ghaffari F. (2021). CNFE-SE: A novel approach combining complex network-based feature engineering and stacked ensemble to predict the success of intrauterine insemination and ranking the features. BMC Med. Inform. Decis. Mak..

[42] Zippl A.L., Wachter A., Rockenschaub P., Toth B., Seeber B. (2022). Predicting success of intrauterine insemination using a clinically based scoring system. Arch. Gynecol. Obstet..

