# Supplementary material for: An Artificial Intelligence-Based Model to Predict Pregnancy After Intrauterine Insemination: A Retrospective Analysis of 9501 Cycles
Source: J Pers Med. 2025 Jul 12;15(7):308. doi: 10.3390/jpm15070308 (PMC12300755; doi:10.3390/jpm15070308)
Supplement: Supplementary file 1 [file jpm-15-00308-s001.zip › jpm-3667688-supplementary.pdf]

SUPPLEMENTAL MATERIAL

Supplemental Table S1: Description of the study dataset features.

ID	Feature	Description	Variable
**1**	Maternal age	Maternal age (in years) at the IUI	Discrete
**2**	Paternal age	Paternal age (in years) at semen collection	Discrete
**3**	Pre-wash sperm concentration	Concentration of the fresh semen specimen prior to washing	Continuous
**4**	Pre-wash sperm A	Percentage of rapid progressive sperm prior to processing	Continuous
**5**	Pre-wash sperm B	Percentage of slow progressive sperm prior to processing	Continuous
**6**	Pre-wash sperm C	Percentage of non-progressive sperm prior to processing	Continuous
**7**	Pre-wash sperm D	Percentage of immotile sperm prior to processing	Continuous
**8**	Pre-wash sperm A+B	Percentage of rapid plus slow progressive sperm prior to processing	Continuous
**9**	Pre-wash sperm A+B+C	Percentage of rapid, slow, and non-progressive sperm prior to processing	Continuous
**10**	Post-wash sperm concentration	Concentration of the sperm after washing and separation from the seminal fluid	Continuous
**11**	Post-wash sperm A	Percentage of rapid progressive sperm after processing	Continuous
**12**	Post-wash sperm B	Percentage of slow progressive sperm after processing	Continuous
**13**	Post-wash sperm C	Percentage of non-progressive sperm after processing	Continuous
**14**	Post-wash sperm D	Percentage of immotile sperm after processing	Continuous
**15**	Post-wash sperm A+B	Percentage of rapid plus slow progressive sperm after processing	Continuous
**16**	Post-wash sperm A+B+C	Percentage of rapid, slow, and non-progressive sperm after processing	Continuous
**17**	NMSI	Number of motile spermatozoa inseminated	Continuous
**18**	IUI history	Number of intrauterine insemination cycles the couples underwent (i.e., 1-5)	Categorical
**19**	Ovarian stimulation protocol	Type of ovarian stimulation protocol prior to IUI (i.e., oral agents only, exogenous gonadotropins only, oral agents combined with exogenous gonadotropins, or natural cycles)	Categorical
**20**	Cycle duration	Number of days between the last menses and the intrauterine insemination	Discrete
**21**	Number of days to the pregnancy test	Number of days between the last menses and the pregnancy test	Discrete

Note: In cases where couples underwent multiple IUI cycles, the cycle duration and number of days to the positive pregnancy test were calculated for each new cycle.

Supplemental Table S2: Distribution of female patients undergoing IUI by age group. The majority of patients were between 30–34 years old (36%), followed by those aged 35–39 years (32%).

Female age (years)	Number of patients (n)	% of patients
**<30**	679	19%
**30-34**	1,281	36%
**35-39**	1,137	32%
**>40**	438	13%

Supplemental Table S3 Performance metrics of various machine learning models for predicting IUI success using all available features and the top 4 selected features. Metrics reported include area under the ROC curve (AUC), recall, and F1 score with corresponding standard deviations based on cross-validation. Linear SVM consistently outperformed other models across both feature sets, particularly in AUC and F1 score.

Feature set	Model	AUC	Recall	F1 Score
All	AdaBoost	0.57 ± 0.03	0.67 ± 0.01	0.53 ± 0.10
	**Linear SVM**	**0.76 ± 0.04**	**0.73 ± 0.01**	**0.76 ± 0.10**
	Kernel SVM	0.56 ± 0.05	0.49 ± 0.02	0.55 ± 0.04
	Random Forest	0.56 ± 0.03	0.65 ± 0.01	0.53 ± 0.02
	Extreme Forest	0.55 ± 0.04	0.69 ± 0.01	0.52 ± 0.01
	Bagging Classifier	0.53 ± 0.05	0.51 ± 0.01	0.49 ± 0.10
	Voting Classifier	0.56 ± 0.05	0.50 ± 0.02	0.55 ± 0.10
Top 4	AdaBoost	0.58 ± 0.05	0.69 ± 0.01	0.56 ± 0.05
	**Linear SVM**	**0.78 ± 0.04**	**0.77 ± 0.02**	**0.78 ± 0.04**
	Kernel SVM	0.57 ± 0.05	0.39 ± 0.01	0.57 ± 0.05
	Random Forest	0.58 ± 0.04	0.56 ± 0.02	0.58 ± 0.01
	Extreme Forest	0.56 ± 0.06	0.55 ± 0.01	0.55 ± 0.05
	Bagging Classifier	0.54 ± 0.04	0.50 ± 0.02	0.53 ± 0.05
	Voting Classifier	0.57 ± 0.05	0.51 ± 0.05	0.57 ± 0.05

Supplemental Table S4 Summary of selected studies evaluating predictive models for intrauterine insemination (IUI) success. The table compares datasets, number of features, analytical methods used, and main limitations.

Main finding	Dataset	Features	Analytical method	Limitations	Ref.
Predictions of IUI outcome	1,438 couples 3,375 IUI cycles	8	Logistic regression analysis	Few features	Goldman et al., 2014
Impact of sperm morphology on IUI success	412 couples 530 IUI cycles	12	Statistical analysis	Few samples	Erdem et al., 2015
IUI outcome based on sperm parameters	1,166 couples 4,251 IUI cycles	9	Multivariable logistic regression	Few features	Lemmens et al., 2016
Prediction of semen impact on IUI success rate	556 couples 1,401 IUI cycles	16	Logistic regression	Small dataset	Thijssen et al., 2017
Effects of FSH and CC in infertile women with unexplained infertility	684 couples 2,259 IUI cycles	8	Logistic regression	Few features	Danhof et al., 2019
Predict IUI success and perform feature selection	8,360 couples, 11,255 IUI cycles	256	Network based feature engineering	Non-characterized patient population	Ranjbari et al., 2021
Prediction using clinically based score system	758 couples, 1437 IUI cycles	Clinical and laboratory features	Multivariable logistic regression	Small dataset	Zippl et al., 2022
Calculator for IUI success	299 couples, 355 IUI cycles	5	Multivariable stepwise regression	Small dataset	Ejzenberg et al., 2025

FSH, follicle stimulating hormone; IUI, intrauterine insemination; CC, clomiphene cytrate

References

Goldman RH, Batsis M, Petrozza JC, Souter I. Patient-specific predictions of outcome after gonadotropin ovulation induction/intrauterine insemination. Fertil Steril. 2014 Jun;101(6):1649-55.

Erdem M, Erdem A, Mutlu MF, Ozisik S, Yildiz S, Guler I, Karakaya C. The impact of sperm morphology on the outcome of intrauterine insemination cycles with gonadotropins in unexplained and male subfertility. Eur J Obstet Gynecol Reprod Biol. 2016 Feb;197:120-4. doi: 10.1016/j.ejogrb.2015.12.014. Epub 2015 Dec 19. PMID: 26745391.

Lemmens L, Kos S, Beijer C, Brinkman JW, van der Horst FA, van den Hoven L, Kieslinger DC, van Trooyen-van Vrouwerff NJ, Wolthuis A, Hendriks JC, Wetzels AM; Semen Section of the Dutch Foundation for Quality Assessment in Medical Laboratories. Predictive value of sperm morphology and progressively motile sperm count for pregnancy outcomes in intrauterine insemination. Fertil Steril. 2016 Jun;105(6):1462-8. doi: 10.1016/j.fertnstert.2016.02.012. Epub 2016 Mar 2. PMID: 26930619.

Thijssen A, Creemers A, Van der Elst W, Creemers E, Vandormael E, Dhont N, Ombelet W. Predictive value of different covariates influencing pregnancy rate following intrauterine insemination with homologous semen: a prospective cohort study. Reprod Biomed Online. 2017 May;34(5):463-472. doi: 10.1016/j.rbmo.2017.01.016. Epub 2017 Feb 24. PMID: 28285953.

Danhof NA, van Eekelen R, Repping S, Mol BWJ, van der Veen F, van Wely M, Mochtar MH; SUPER Study group. Follicle stimulating hormone or clomiphene citrate in intrauterine insemination with ovarian stimulation for unexplained subfertility: a role for treatment selection markers? Reprod Biomed Online. 2019 Jun;38(6):938-942. doi: 10.1016/j.rbmo.2019.01.014. Epub 2019 Mar 2. PMID: 30981620.

Ranjbari S, Khatibi T, Vosough Dizaji A, Sajadi H, Totonchi M, Ghaffari F. CNFE-SE: a novel approach combining complex network-based feature engineering and stacked ensemble to predict the success of intrauterine insemination and ranking the features. BMC Med Inform Decis Mak. 2021 Jan 2;21(1):1. doi: 10.1186/s12911-020-

Zippl AL, Wachter A, Rockenschaub P, Toth B, Seeber B. Predicting success of intrauterine insemination using a clinically based scoring system. Arch Gynecol Obstet. 2022 Nov;306(5):1777-1786. doi: 10.1007/s00404-022-06758-z. Epub 2022 Sep 7. PMID: 36069921; PMCID: PMC9519724.

Ejzenberg D, Callado GY, de Oliveira Gomes TJ, Cavalcanti GS, Soares JM Jr, Baracat EC, Monteleone PA. A new accurate model to assess intrauterine insemination success based on clinical parameters: Optimizing fertility treatment. Int J Gynaecol Obstet. 2025 Apr 2. doi: 10.1002/ijgo.70104. Epub ahead of print. PMID: 40172155.
